# The association of germline variants with chronic lymphocytic leukemia outcome suggests the implication of novel genes and pathways in clinical evolution

**DOI:** 10.1186/s12885-019-5628-y

**Published:** 2019-05-29

**Authors:** Adrián Mosquera Orgueira, Beatriz Antelo Rodríguez, Natalia Alonso Vence, José Ángel Díaz Arias, Nicolás Díaz Varela, Manuel Mateo Pérez Encinas, Catarina Allegue Toscano, Elena María Goiricelaya Seco, Ángel Carracedo Álvarez, José Luis Bello López

**Affiliations:** 1 Clinical University Hospital of Santiago de Compostela, Service of Hematology and Hemotherapy, 1st floor, Avenida da Choupana s/n, Santiago de Compostela, 15706 Spain; 2 Division of Hematology, SERGAS, Complexo Hospitalario Universitario de Santiago de Compostela (CHUS), Santiago, Spain; 3 University of Santiago de Compostela, Santiago, Spain; 4 Fundación Pública de Medicina Xenómica, A Coruña, Spain

**Keywords:** Chronic lymphocytic leukemia, Germline, Polymorphism, Association, Prognosis

## Abstract

**Background:**

Chronic Lymphocytic Leukemia (CLL) is the most frequent lymphoproliferative disorder in western countries and is characterized by a remarkable clinical heterogeneity. During the last decade, multiple genomic studies have identified a myriad of somatic events driving CLL proliferation and aggressivity. Nevertheless, and despite the mounting evidence of inherited risk for CLL development, the existence of germline variants associated with clinical outcomes has not been addressed in depth.

**Methods:**

Exome sequencing data from control leukocytes of CLL patients involved in the International Cancer Genome Consortium (ICGC) was used for genotyping. Cox regression was used to detect variants associated with clinical outcomes. Gene and pathways level associations were also calculated.

**Results:**

Single nucleotide polymorphisms in *PPP4R2* and *MAP3K4* were associated with earlier treatment need. A gene-level analysis evidenced a significant association of *RIPK3* with both treatment need and survival. Furthermore, germline variability in pathways such as apoptosis, cell-cycle, pentose phosphate, GNα13 and Nitric oxide was associated with overall survival.

**Conclusion:**

Our results support the existence of inherited conditionants of CLL evolution and points towards genes and pathways that may results useful as biomarkers of disease outcome. More research is needed to validate these findings.

**Electronic supplementary material:**

The online version of this article (10.1186/s12885-019-5628-y) contains supplementary material, which is available to authorized users.

## Background

Chronic Lymphocytic Leukemia (CLL) is the most frequent lymphoproliferative disease in western countries, and it shows remarkable clinical heterogeneity [1]. Recently, some studies demonstrated a wealth of genomic and epigenomic differences that determine part of its clinical aggressivity [2], such as point mutations in *NOTCH1*, *SF3B1*, *ATM*, *TP53* and *POT1*, and the absence of somatic hypermutation in the *IGHV* locus.

Inherited predisposition to the development of CLL has been addressed by various genome wide association studies (GWAS) during the last years. In this regard, dozens of common variants at genes such as *BCL2*, *EOMES*, *CASP10* and *POT1* have been associated with significant risk of CLL development [3–5]. Similarly, GWAS studies in other lymphoproliferative disorders such as follicular lymphoma and diffuse large B cell lymphoma have found evidence for the association of germline variants with overall survival (OS) and progression-free survival [6, 7]. Despite this evidence, analysis of CLL clinical evolution have been limited almost exclusively to acquired somatic events.

In this paper, we addressed for the first time to our knowledge the association of common genomic variants with time to treatment (TTT) and OS of CLL patients participating in the Spanish ICGC cohort. Our results suggest the existence of polymorphisms at some genes (e.g., *PPP4R2* and *MAP3K4*) significantly associated with TTT. Moreover, we found significant associations with TTT and OS both at the gene and pathway-level, which could shed new light about CLL biology and its mechanisms of progression.

## Methods

### Data source

We applied for access to the *International Cancer Genome Consortium* (ICGC) CLL sequencing data [8] deposited in the *European Genome-Phenome Database* (EGA). The Data Access Committee approved access to this data under *DACO-1040945*. We downloaded exome-seq data from control non-tumoral samples from patients with CLL under the accession code *EGAD00001001464*.

### Data preprocessing

Exome-seq data were previously aligned to the reference genome (GRCh37.75) using *bwa* [9] as described in *Puente* et al [10]. Briefly, 3 μg of genomic DNA were used for paired-end sequencing library construction, followed by enrichment in exomic sequences using the *SureSelect Human All Exon 50 Mb v4* kit or the *SureSelect Human All Exon 50 Mb + UTR* kits (Agilent Technologies). Next, DNA was pulled down using magnetic beads with streptavidin, followed by 18 cycles of amplification. Sequencing was performed on an Illumina GAIIx or on a HiSeq2000 sequencer (2x76bp). Duplicate read removal, sorting and indexing was done using *samtools* [11]. Base quality score recalibration was made with *BamUtil* [12] using a logistic regression model.

### Variant detection and filtering

Platypus2 [13] was run on genotyping mode. All dbSNP variants [14] were used as input for genotyping. We used the following specifications: “*minVarFreq = 0.02*”, “*minReads = 2*”, “*maxReads = 8000*”, “*assemble = 1*”, “*minBaseQual = 20*”, “*trimSoftClipped = 1*”, “*minPosterior = 20*”, “*sbThreshold = 0.01*”, “*badReadsWindow = 15*” and “*badReadsThreshold = 15*”, at least 10 reads covering a position and 2 reads covering a variant, a minimum genotype quality (GQ) of 20 Phred, genotype likelihood (GL) below − 3, maximum homopolymer run (HP) below 11, minimum variant quality adjusted per read depth (QD) above 2 and minimum median minimum base quality for bases around variant (MMLQ) above 10. Variants labeled by platypus as “*HapScore*”, “*SC*”, “*strandBias*” and “*MQ*” were discarded. Heterozygous loci with variant allele frequency (VAF) < 35% or > 70% were also discarded.

### Sample filtering

We used principal component analysis (PCA) to detect outliers in our study cohort. Similarly, identity-by-descent (IBD) was used to discard all individuals with a degree of relatedness equivalent to third degree or higher. PCAs and IBD data were computed on a linkage disequilibrium (LD) pruned dataset (LD upper threshold of 0.2) using the *Bioconductor* [15] package *SNPRelate* [16]. Our final filtered dataset contained 426 cases. Among these, 253 were males and 173 were females. By *IGHV* status, there were 146 unmutated cases and 273 mutated cases; and by clinical staging, the data contained 47 monoclonal B-cell lymphocytosis (MBL), 332 Binet A, 37 Binet B and 8 Binet C cases. Information about clinical staging and *IGHV* mutation status was not available for 2 and 7 cases, respectively.

### Regression analysis

Cox regression and assumption of proportional hazards was performed with the *survival* R package [17, 18]. Variables with *p*-value < 0.2 in a univariate model were selected as covariates for the GWAS. In the cases of TTT these were *“donor sex”*, *“IGHV mutation status”* and *“Binet stage”*; whilst in the case of overall survival we used *“IGHV mutation status”*, *“Binet stage”* and *“donor age at diagnosis”* as covariates. Three association models were computed: an additive model, a dominant model and a recessive model. *P*-values were adjusted using the Benjamini-Hochberg (BH) method.

Due to the heterogeneity of exome-seq coverage and quality metrics, many variables had incomplete data. We included in the analysis variables with at least 25% call rate, a minimum of 10 events (progression or death). A minimum allele frequency of 1% was selected as the lowest threshold. Furthermore, we only analyzed polymorphisms where Platypus called at least 10 minor alleles (additive model) or genotypes (dominant and recessive models).

Inflation values were estimated with the R package *QQperm* [19]. Briefly, a random distribution of *p*-values was created by randomly permuting phenotype variables. Then, the association p-values are compared with the null. This method doesn’t consider the null distribution to be distributed uniformly.

### Gene-level analysis

*VEGAS2* [20] was used to calculate LD-adjusted association p-values for TTT and OS. Briefly, *VEGAS2* takes GWAS p-values, and then uses a simulation-based approach using information from population variant reference panels to adjust for LD effects. We used the 1000 Genomes phase 3 data from the iberian population in Spain as our reference population, since all patients of this cohort were of Spanish origin [21]. Only variants falling within the 5′ and 3′ coordinates of RefSeq genes were included. *P*-values were adjusted for multiple testing using the BH method.

### Pathways and gene ontology (GO) analysis

*GSEA4GWAS* version 1.1 [22] was used for testing significant associations in pathways and biological process annotations. Our input pathways were *“Canonical Pathways”* and *“Gene Ontology Biological Process”*. Maximum distance was set to 20 Kb, and the major histocompatibility complex region was masked from the analysis. P-values were adjusted with the BH method.

## Results

### Genomic polymorphisms associated with treatment-free survival

**Fig. 1:**
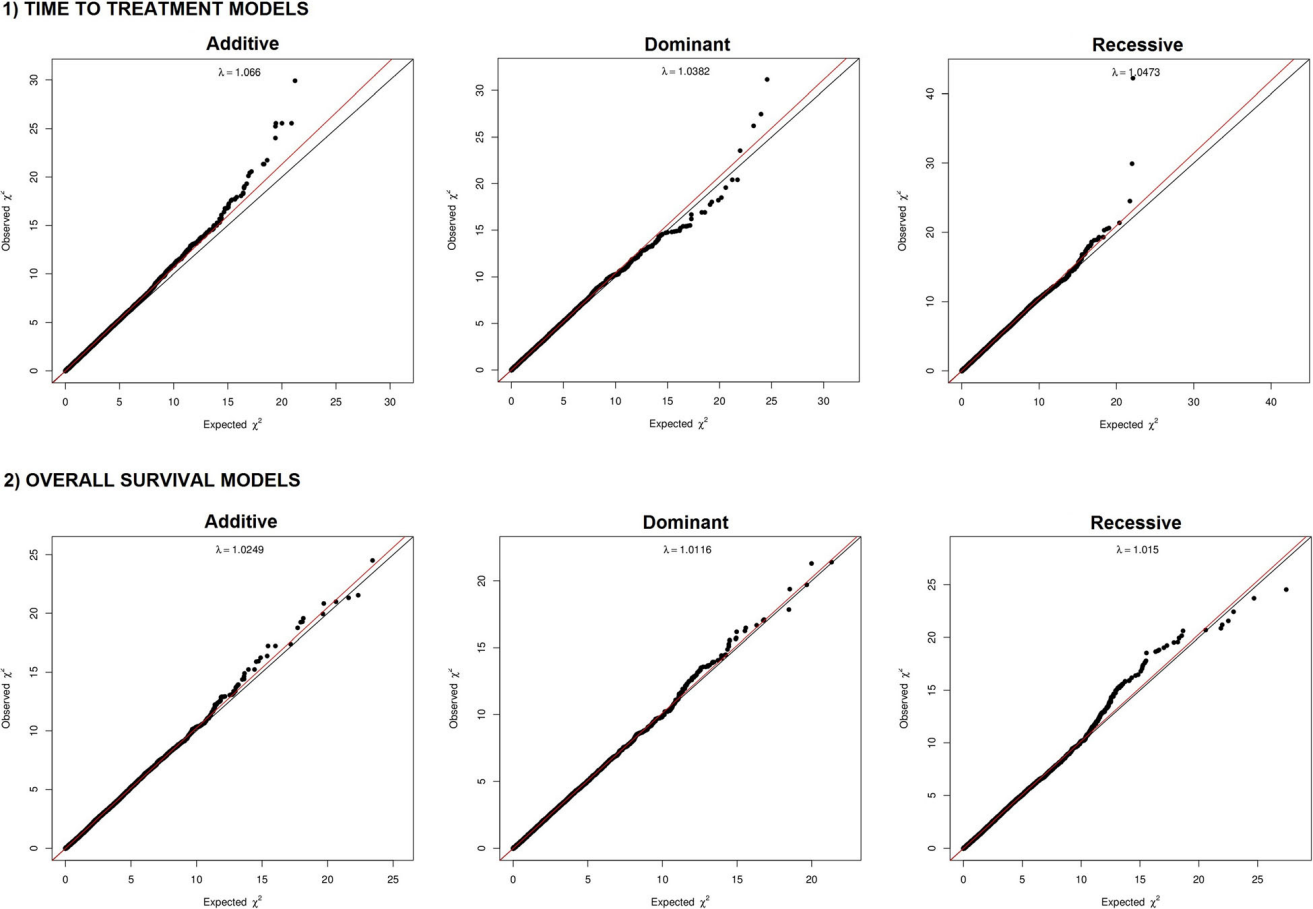
QQplots for the TTT and OS additive, dominant and recessive models

**Fig. 2:**
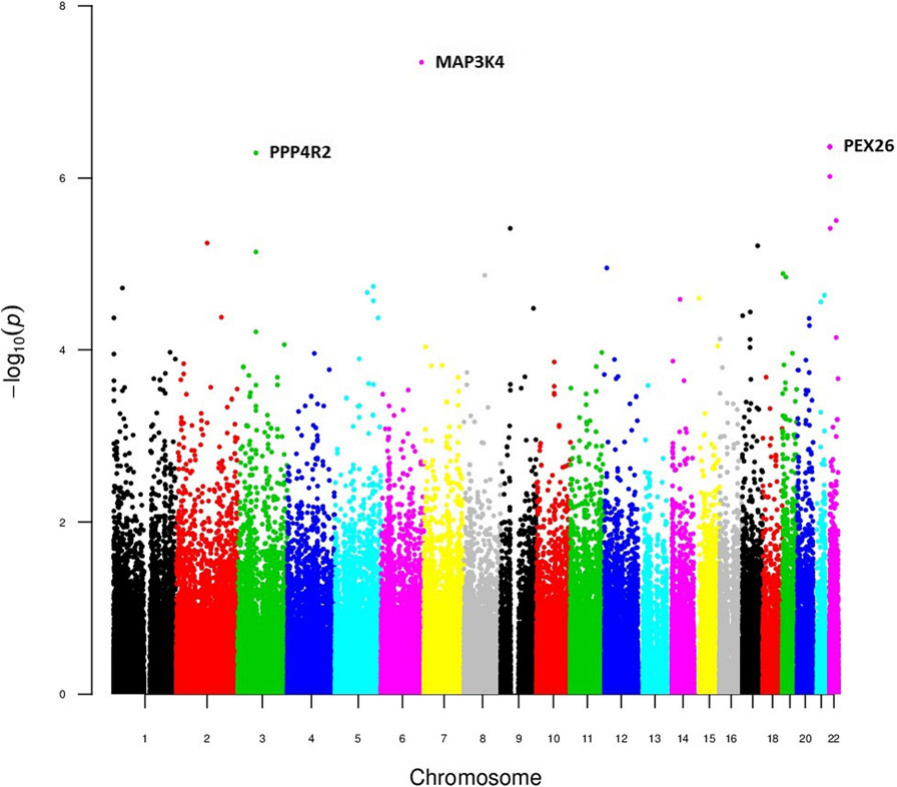
Manhattan plot of the additive TTT model results

We created three models to analyze variant association with time to first treatment: an additive, a dominant and a regressive model. PCA plots (Additional file 1: Figure S1) and lambda inflation values (lambda values of 1.06, 1.03 and 1.04, Fig. 1) revealed no significant inflation or population stratification. In the additive model we observed 6 polymorphisms associated with TTT (BH adjusted *p*-value < 0.05), and other 6 showed association with BH adjusted p-value in the range of 0.05–0.1 (Fig. 2, Table 1, Additional file 9 Table S1). These variants were located in *MAP3K4*, *PEX26* (4 variants), *PPP4R2* (2 variants), *TTLL12/TSPO*, *TXNRD2*, *ZCCHC7*, *MKI67IP* and *MARCH10*. Notably, rs537453728 at *MAP3K4* broke the genome-wide association p-value (4.53 × 10^− 8^). Other 391 variants were suggestively associated with TTT (BH adjusted *p*-value 0.1–0.5).

**Table 1:** Results of the additive, dominant and recessive TTT models. Polymorphisms with a BH-adjusted *P*-value < 0.1 are show

rs ID	*P*-value	BH-adjusted *P*-value	Reference	Alternative	Gene Symbol	Hazard Ratio	MAF
ADDTIVE MODEL							
*rs537453728*	4.53E-08	5.39 E-3	CT	C	*MAP3K4*	7.08	0.01
*rs361807*	4.31E-07	0.01	T	C	*PEX26*	4.55	0.1
*rs361946*	4.31E-07	0.01	A	G	*PEX26*	4.55	0.1
*rs1043278*	4.31E-07	0.01	A	G	*PEX26*	4.55	0.1
*rs7620924*	5.08E-07	0.01	A	G	*PPP4R2*	0.24	0.49
*rs5992169*	9.55E-07	0.02	G	A	*PEX26*	4.45	0.11
*rs9463*	3.11E-06	0.05	G	A	*TTLL12*	3.18	0.42
*rs55715863*	3.84E-06	0.05	C	A	*TXNRD2*	5.24	0.01
*rs3780333*	3.84E-06	0.05	A	G	*ZCCHC7*	4.8	0.27
*rs17016977*	5.69E-06	0.07	A	G	*MKI67IP*	5.53	0.01
*rs72842201*	6.13E-06	0.07	A	G	*MARCH.10*	2.99	0.05
*rs3172278*	7.23E-06	0.07	T	C	*PPP4R2*	4.04	0.49
DOMINANT MODEL							
*rs3172278*	2.40E-08	1.23E-03	T	C	*PPP4R2*	0.09	0.49
*rs2247870*	1.60E-07	4.10E-03	G	A	*GPR98*	0.4	0.47
*rs9463*	3.07E-07	5.26E-03	G	A	*TTLL12*	0.17	0.42
*rs28656102*	1.22E-06	0.02	T	C	*PPP4R2*	0.18	0.42
*rs1045960*	6.22E-06	0.05	C	T	*PPP4R2*	0.21	0.42
*rs62039297*	6.29E-06	0.05	G	A	*SRL*	0.2	0.26
*rs9873229*	9.72E-06	0.07	C	T	*PPP4R2*	0.22	0.44
RECESSIVE MODEL							
*rs7620924*	8.07E-11	1.08E-05	A	G	*PPP4R2*	11.77	0.49
*rs537453728*	4.53E-08	3.04E-03	CT	C	*MAP3K4*	0.14	0.01
*rs9310254*	7.49E-07	0.03	T	C	*PPP4R2*	5.56	0.49

**Fig. 3:**
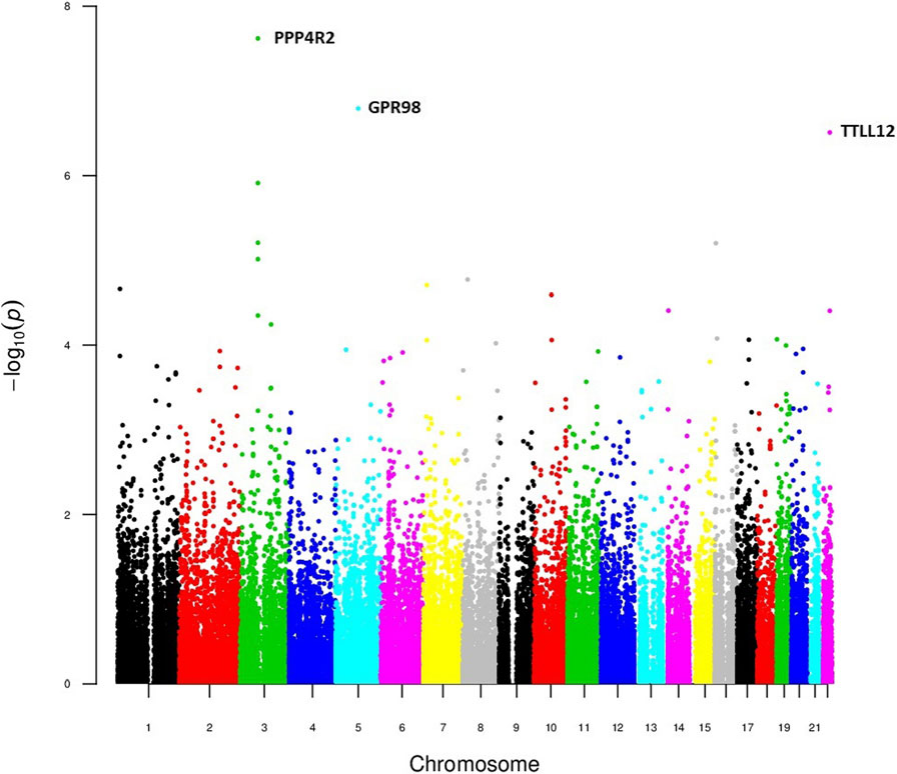
Manhattan plot of the dominant TTT model results

**Fig. 4:**
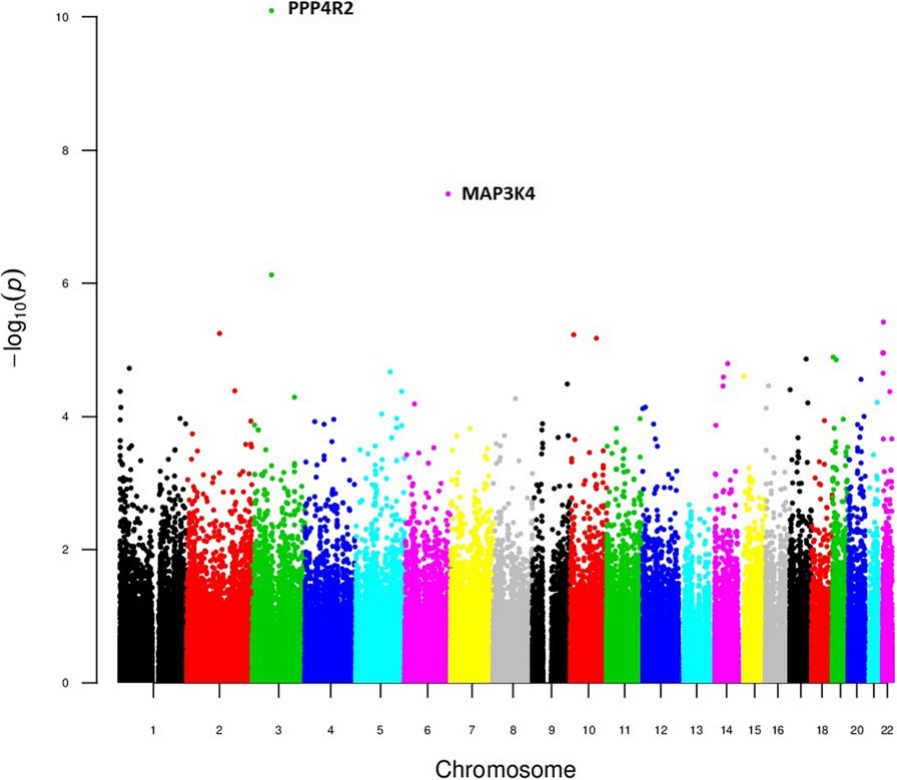
Manhattan plot of the recessive TTT model result

The dominant and the recessive models evidenced very significant enrichment of variants at *PPP4R2* (lowest p-value at rs7620924: 8.07 × 10^− 11^). Variants at *GPR98*, *MAP3K4* and *TTLL12* were also significantly associated with TTT (BH adjusted *p*-value < 0.05) (Figs. 3 and 4, Table 1, Additional file 9: Tables S2 and S3).

Some of this polymorphisms are associated with functional changes in their corresponding genes. According to dbSNP [14], rs2247870 induces a missense change in the *GPR98* gene. In a similar fashion, a search in the HaploReg database [23] points toward functional implications of some of these variants. For example, rs7620924 is located in a lymphocyte-specific enhancer region that is strongly associated with *PPP4R2* expression in whole blood (*p*-value 2.18 × 10^− 34^) and in lymphoblastoid cells (*p*-value 2.36 × 10^− 8^). Similarly, the polymorphisms rs361807, rs361946, rs5992169 and rs1043278 in *PEX36* are associated with *PEX36* expression in lymphoblastoid cells (minimum p-value 6.66 × 10^− 10^). rs9463 in *TTLL12* strongly correlates with the expression of the adjacent gene *TSPO* (p-value 9.81 × 10^− 198^), and to a lower extent, with *TTLL12* (p-value 9.57 × 10^− 4^) in blood cells. Other polymorphisms suggestively associated with TTT such as those in *TXNRD2* and *ZCCHC7* are also significantly associated with the expression of their respective genes in blood cells. On the contrary, no functional information exists about the intronic variant rs537453728 within the *MAP3K4* gene.

### Genomic polymorphisms associated with overall survival

We created an additive, a dominant and a recessive model to investigate variant association with OS. No significant inflation was observed (lambda values of 1.02, 1.01 and 1.01 for additive, dominant and recessive models, respectively). No variant achieved BH-adjusted *p*-values below 0.05 in any model (Additional file 2: Figure S2, Additional file 3: Figure S3 Additional file 4: Figure S4, Table 2, Additional file 9: Tables S4-S6). Nevertheless, 20 variants were below 0.06 in the additive models, with the top variants falling in *TTC32*, *WDR35* and *CLIP1*. Notably, rs2304588 at *TTC32* achieved the lowest *p*-value (7.6 × 10^− 7^, Bonferroni-adjusted p-value 0.067, BH-adjusted p-value 0.056). Overall, the number of variants with BH adjusted *p*-values below 0.1 was 25 in the additive model, 4 in the dominant model and 18 in the recessive model.

**Table 2:** Results of the additive, dominant and recessive OS models. Polymorphisms with a BH-adjusted *P*-value < 0.1 are show

rs ID	*P*-value	BH-adjusted *P*-value	Reference	Alternative	Symbol	Hazard Ratio	MAF
ADDITIVE							
*rs2304588*	7.36E-07	0.06	A	G	*TTC32*	6.59	0.08
*rs2293671*	1.43E-06	0.06	T	C	*WDR35*	4.97	0.08
*rs139689179*	2.17E-06	0.06	G	C	*CLIP1*	33.51	0.02
*rs149301745*	3.45E-06	0.06	ACTT	A	*SPATA5L1*	6.04	0.04
*rs56127964*	3.88E-06	0.06	A	G	*ACO1*	7.51	0.02
*rs1800502*	4.65E-06	0.06	A	G	*CFTR*	6.23	0.024
*rs1060742*	4.88E-06	0.06	T	C	*WDR35*	4.05	0.08
*rs72860063*	4.98E-06	0.06	A	G	*KIF6*	11.19	0.02
*rs7563410*	6.46E-06	0.06	C	A	*TTC32*	3.97	0.08
*rs28502265*	6.48E-06	0.06	T	G	*WDR35*	3.98	0.08
*rs769479742*	7.23E-06	0.06	T	G	*PDE1C*	7.39	0.03
*rs76774695*	7.96E-06	0.06	C	T	*PARP6*	10.54	0.01
*rs148915854*	8.41E-06	0.06	T	TA	*FAM184B*	5.19	0.07
*rs114314465*	9.74E-06	0.06	A	C	*SLC4A5*	10.69	0.02
*rs3732783*	9.77E-06	0.06	T	C	*DRD3*	13.34	0.09
*rs117149407*	9.79E-06	0.06	G	A	*EYA1*	14.24	0.03
*rs12194408*	1.14E-05	0.06	C	G	*PPIL1*	7.15	0.02
*rs1790557*	1.16E-05	0.06	C	T	*TENM4*	7.98	0.25
*rs35034822*	1.27E-05	0.06	T	G	*PROM2*	14.77	0.02
*rs147203128*	1.29E-05	0.06	G	A	*CYTH2*	12.94	0.02
*chr2:95944632*	1.42E-05	0.06	T	G	*PROM2*	14.47	0.02
*rs200619224*	1.49E-05	0.06	A	C	*CDC23*	6.15	0.02
*rs75827446*	1.57E-05	0.06	T	C	*TNL2*	43.14	0.02
*rs1053361*	1.68E-05	0.06	G	C	*RHCE*	11.75	0.02
*rs7270676*	2.44E-05	0.09	T	C	*SEMG1*	6.84	0.04
DOMINANT							
*rs7512589*	3.71E-06	0.07	G	A	*CELA2A*	0.21	0.29
*rs3737697*	3.93E-06	0.07	C	T	*CELA2A*	0.21	0.29
*rs62039297*	9.09E-06	0.1	G	A	*SRL*	0.06	0.26
*rs10783474*	1.08E-05	0.1	G	A	*SCN8A*	0.11	0.16
RECESSIVE							
*rs2304588*	7.36E-07	0.05	A	G	*TTC32*	0.15	0.08
*rs2293671*	1.12E-06	0.05	T	C	*WDR35*	0.18	0.08
*rs139689179*	2.17E-06	0.06	G	C	*CLIP1*	0.03	0.02
*rs149301745*	3.45E-06	0.06	ACTT	A	*SPATA5L1*	0.17	0.04
*rs1060742*	4.18E-06	0.06	T	C	*WDR35*	0.23	0.08
*rs72860063*	4.98E-06	0.06	A	G	*KIF6*	0.09	0.02
*rs7563410*	5.41E-06	0.06	C	A	*TTC32*	0.24	0.08
*rs28502265*	5.59E-06	0.06	T	G	*WDR35*	0.24	0.08
*rs769479742*	7.23E-06	0.07	T	G	*PDE1C*	0.14	0.03
*rs76774695*	7.96E-06	0.07	C	T	*PARP6*	0.09	0.01
*rs114314465*	9.74E-06	0.08	A	C	*SLC4A5*	0.09	0.02
*rs117149407*	9.79E-06	0.08	G	A	*EYA1*	0.07	0.03
*rs12194408*	1.14E-05	0.08	C	G	*PPIL1*	0.14	0.02
*rs35034822*	1.27E-05	0.08	T	G	*PROM2*	0.07	0.02
*chr2:95944632*	1.42E-05	0.08	T	G	*PROM2*	0.07	0.02
*rs200619224*	1.49E-05	0.08	A	C	*CDC23*	0.16	0.02
*rs75827446*	1.57E-05	0.08	T	C	*TLN2*	0.02	0.02
*rs1053361*	1.68E-05	0.09	G	C	*RHCE*	0.09	0.02

### Gene-based association with TTT and OS

Gene-level integration of *p*-values for the TTT model using *VEGAS2* evidenced the association of 5 genes with BH adjusted *p*-values < 0.05 and 10 genes with BH adjusted p-values in the range of 0.05–0.1 (Table 3, Additional file 9: Table S7). The most significant genes were *AURKAIP1*, *NIFK*, *RIPK3*, *SIK1* and *ZCCHC7*.

**Table 3:** *VEGAS2* gene-level analysis of the additive TTT and OS models. Genes with a BH-adjusted *P*-value < 0.1 are shown

Gene	nSNPs	nSims	Test	*P* value	TopSNP	TopSNP.*p* value	BH-ajdusted *p*-value
VEGAS2 Analysis of TTT							
*AURKAIP1*	2	1.00E+06	26.72	1.00E-06	*rs2765035*	2.28E-04	6.90E-03
*NIFK*	4	1.00E+06	36.22	1.00E-06	*rs17016977*	5.69E-06	6.90E-03
*RIPK3*	7	1.00E+06	22.32	2.00E-06	*rs28379107*	0.02	9.20E-03
*SIK1*	3	1.00E+06	31.07	9.00E-06	*rs3746951*	2.32E-05	0.03
*ZCCHC7*	2	1.00E+06	23.2	1.10E-05	*rs3780333*	3.84E-06	0.03
*ABCA7*	18	1.00E+06	88.04	2.30E-05	*rs3752230*	3.22E-04	0.05
*PEX26*	7	1.00E+06	83.49	2.70E-05	*rs361807*	4.31E-07	0.05
*AARS*	3	1.00E+06	17.21	4.10E-05	*rs2070203*	2.34E-03	0.06
*NIFK-AS1*	3	1.00E+06	24.72	4.50E-05	*rs17016977*	5.69E-06	0.06
*TSPO*	2	1.00E+06	24.36	4.60E-05	*rs6971*	7.18E-05	0.06
*MARCH.10*	2	1.00E+06	20.45	4.80E-05	*rs72842201*	6.13E-06	0.06
*LOC574538*	3	1.00E+06	24.15	7.20E-05	*rs56204927*	1.94E-04	0.08
*SEC24D*	10	1.00E+06	44.69	7.60E-05	*rs115446044*	1.09E-03	0.08
*TTLL12*	9	1.00E+06	54.06	9.90E-05	*rs9463*	3.11E-06	0.1
*GALNT11*	4	1.00E+06	15.18	1.07E-04	*rs146169444*	0.01	0.1
VEGAS2 Analysis of OS							
*CLUAP1*	7	1.00E+06	57.76	1.00E-06	*rs78851263*	3.27E-04	6.17E-03
*TTC32*	5	1.00E+06	66.51	1.00E-06	*rs2304588*	7.36E-07	6.17E-03
*DRD3*	2	1.00E+06	21.54	4.00E-06	*rs3732783*	9.77E-06	0.02
*RAD51AP2*	7	1.00E+06	22.31	6.00E-06	*rs62130401*	0.02	0.02
*RIPK3*	8	1.00E+06	18.64	1.30E-05	*rs3212251*	0.01	0.03
*EPYC*	2	1.00E+06	17.78	2.00E-05	*rs76171854*	3.25E-04	0.03
*TENM4*	13	1.00E+06	75.05	2.10E-05	*rs1790557*	1.16E-05	0.03
*GTPBP1*	6	1.00E+06	17.81	2.30E-05	*rs16999297*	6.42E-03	0.03
*GAMT*	2	1.00E+06	17.56	2.80E-05	*rs266809*	8.65E-05	0.03
*DCP1B*	16	1.00E+06	61.9	3.00E-05	*rs150660202*	7.13E-03	0.03
*MIR548D1*	3	1.00E+06	17.35	3.30E-05	*rs12141159*	4.48E-04	0.03
*MIR548AA1*	3	1.00E+06	17.35	3.40E-05	*rs12141159*	4.48E-04	0.03
*WDR35*	14	1.00E+06	89.29	5.90E-05	*rs2293671*	1.43E-06	0.06
*BNIPL*	6	1.00E+06	23.11	7.40E-05	*rs955955*	2.91E-03	0.06
*KARS*	3	1.00E+06	15.43	1.05E-04	*rs148298278*	1.28E-04	0.09
*ACO1*	13	1.00E+06	57.84	1.26E-04	*rs56127964*	3.88E-06	0.1
*PARP6*	3	1.00E+06	21.16	1.33E-04	*rs76774695*	7.96E-06	0.1

Similarly, we observed 12 genes associated with OS at a BH-adjusted *p*-value < 0.05 (Table 2, Additional file 9: Table S8), namely *CLUAP1*, *TTC32*, *DRD3*, *RAD51AP2*, *RIPK3*, *EPYC*, *TENM4*, *GTPBP1*, *GAMT*, *DCP1B*, *MIR548D1* and *MIR548AA1*. Other 5 genes were associated with OS with BH-adjusted *p*-values in the range of 0.05–0.1. These genes were *WDR35*, *BNIPL*, *KARS*, *ACO1* and *PARP6*.

### Pathway-level variation significantly associated with TTT and OS

We used *GSEA4GWAS* to analyze associations with TTT and OS at the pathway level. No significant enrichment neither in *“Canonical pathways”* nor in *“Biological Process”* terms was observed for the TTT variable. Nevertheless, a different scenario was found for the OS variable. Significant *“Canonical pathways”* annotations (BH-adjusted *p*-values < 0.05) were *“Pentose Phosphate pathway”*, *“Gluconeogenesis”*, *“Glycolysis”*, *“GN*α*13 pathway”*, *“Nitric Oxide pathway”*, *“Apoptosis”*, *“Glycolysis and Gluconeogenesis”*, *“Tumor Necrosis Factor pathway”*, *“Ovarian Infertility genes”*, *“Bile Acid Biosynthesis”*, *“Keratinocyte pathway”* and *“Glycine Serine and Threonine pathway”* (Table 4, Additional file 5: Figure S5 and Additional file 6: Figure S6). Other pathways had evidence of suggestive association (BH-adjusted *p*-value < 0.25), such as *“Notch Signalling pathway”*, *“EGF pathway”*, *“JNK MAPK pathway”* and *“PDGF pathway”* among others. Among *“GO Biological Processes”* terms, the following were associated with OS with a BH-adjusted *p*-value < 0.05: *“Induction of Apoptosis by Extracellular Signals”*, *“Mitosis”*, *“M phase”*, *“M phase of mitotic cell cycle”* and *“Negative regulation of Developmental Process”*. Terms with BH-adjusted *p*-value < 0.25 were *“Anti Apoptosis”*, *“Chromosome Segregation”*, *“Vasculature Development”* and *“Negative Regulation of Apoptosis”*, among others (Table 4, Additional file 7: Figure S7 and Additional file 8: Figure S8).

**Table 4:** *GSEA4GWAS* analysis of the additive OS model. *Gene Ontology Biological Process* and *Canonical Pathways* terms with a BH-adjusted *P*-value < 0.25 are shown

Pathway/Gene set name	*P-value*	FDR	Significant genes/Selected genes/All genes
GSEA4GWAS Biological Process results for the Additive OS model			
INDUCTION OF APOPTOSIS BY EXTRACELLULAR SIGNALS	< 0.001	0.01	9/21/27
MITOSIS	< 0.001	0.02	23/68/82
M PHASE	< 0.001	0.02	31/96/114
M PHASE OF MITOTIC CELL CYCLE	< 0.001	0.04	23/70/85
NEGATIVE REGULATION OF DEVELOPMENTAL PROCESS	0.001	0.05	36/148/197
ANTI APOPTOSIS	< 0.001	0.05	20/83/118
CHROMOSOME SEGREGATION	0.003	0.1	10/29/32
CELL CYCLE PHASE	0.001	0.13	38/144/170
CELL CYCLE PROCESS	< 0.001	0.19	42/160/193
REGULATION OF MITOSIS	0.004	0.19	11/36/41
VASCULATURE DEVELOPMENT	0.007	0.24	14/43/55
SKELETAL DEVELOPMENT	0.011	0.25	23/80/103
GAMETE GENERATION	0.011	0.25	18/76/114
NEGATIVE REGULATION OF APOPTOSIS	0.008	0.25	21/112/150
NEGATIVE REGULATION OF PROGRAMMED CELL DEATH	0.008	0.25	21/112/151
GSEA4GWAS Cannonical Pathways results for the Additive OS model			
PENTOSE PHOSPHATE PATHWAY	< 0.001	1.67E-03	8/19/25
GLUCONEOGENESIS	< 0.001	2.00E-03	18/47/53
GLYCOLYSIS	< 0.001	2.00E-03	18/47/53
HSA00030 PENTOSE PHOSPHATE PATHWAY	< 0.001	0.01	8/21/26
ST GA13 PATHWAY	< 0.001	0.02	11/29/37
NO1PATHWAY	< 0.001	0.03	11/26/31
HSA04210 APOPTOSIS	0.001	0.03	19/68/84
HSA00010 GLYCOLYSIS AND GLUCONEOGENESIS	0.001	0.03	17/58/64
ST TUMOR NECROSIS FACTOR PATHWAY	< 0.001	0.04	7/20/29
OVARIAN INFERTILITY GENES	0.001	0.04	8/21/25
BILE ACID BIOSYNTHESIS	0.002	0.04	9/23/27
KERATINOCYTEPATHWAY	0.001	0.04	12/37/46
GLYCINE SERINE AND THREONINE METABOLISM	0.006	0.05	9/28/37
BREAST CANCER ESTROGEN SIGNALING	0.001	0.06	21/78/101
GLYCEROLIPID METABOLISM	0.008	0.09	13/36/45
NOS1PATHWAY	0.01	0.1	7/20/22
HSA00120 BILE ACID BIOSYNTHESIS	0.003	0.11	11/36/38
HSA04330 NOTCH SIGNALING PATHWAY	0.011	0.11	10/35/47
FRUCTOSE AND MANNOSE METABOLISM	0.01	0.11	7/21/25
EGFPATHWAY	0.009	0.11	8/24/27
HSA05218 MELANOMA	0.01	0.11	13/58/71
ST JNK MAPK PATHWAY	0.011	0.12	10/33/40
PDGFPATHWAY	0.011	0.12	8/24/27
TYROSINE METABOLISM	0.015	0.12	8/24/32
HSA05223 NON SMALL CELL LUNG CANCER	0.009	0.12	13/47/54
HSA00760 NICOTINATE AND NICOTINAMIDE METABOLISM	0.019	0.14	7/20/24
HSA00051 FRUCTOSE AND MANNOSE METABOLISM	0.013	0.14	10/35/42
BUTANOATE METABOLISM	0.017	0.14	7/26/29
HSA00260 GLYCINE SERINE AND THREONINE METABOLISM	0.018	0.15	8/36/45
HSA00561 GLYCEROLIPID METABOLISM	0.022	0.19	15/49/58
STRIATED MUSCLE CONTRACTION	0.023	0.2	11/31/39
PPARAPATHWAY	0.023	0.2	11/43/57
HSA04510 FOCAL ADHESION	0.015	0.2	53/171/200
G1PATHWAY	0.04	0.21	4/22/28
HSA05222 SMALL CELL LUNG CANCER	0.024	0.21	22/78/87
HSA05010 ALZHEIMERS DISEASE	0.029	0.21	7/23/28
HIVNEFPATHWAY	0.029	0.22	13/46/58
HSA04662 B CELL RECEPTOR SIGNALING PATHWAY	0.041	0.23	15/57/64
HSA05214 GLIOMA	0.035	0.24	14/56/64
NFATPATHWAY	0.039	0.24	11/46/53

## Discussion

In this work we present evidence that suggest the existence of germline variation modulating CLL’s clinical aggressivity. The most remarkable finding was the strong and recessive association of rs7620924 near *PPP4R2* with short time to treatment. The implication of *PPP4R2* in the regulation of cell survival and DNA repair in hematopoietic and leukemia cells has been recently reported [24]. Indeed, different studies have identified *PPP4R2* as a modulator of protein phosphatase 4 (PPP4), which regulates DNA repair through non-homologous end joining [25]. Concordantly, the ablation of PPP4 activity in mice increases genomic instability and aborgates class switch recombination in B cells, leading to an abnormal immune response [26]; and its function also seems to be essential in V(D) J recombination during normal B cell maturation [27]. Other polymorphisms associated with time to first treatment were located in *MAP3K4*, *PEX26* and *TTLL12*. *MAP3K4* participates in the TRAIL/MAP3K4/p38/HSP27/Akt pathway, thereby modulating processes such as autophagy and cell migration. Indeed, *MAP3K4* is affected by recurrent loss-of-function mutations in different types of cancers [28–32]. Conversely, less is known about the peroxisome-related gene *PEX26* [33]; the G-protein *GPR98*; and *TTLL12*, which participates in chromosome stability and mitosis-related processes [34]. On the contrary, although we did not find any variant significantly associated with overall survival, we devised some variants with suggestive associations. The two most significant ones were in the *TTC32/WDR35* and *CLIP1* loci, the last of which is overexpressed in Reed-Sternberg cells of Hodgkin lymphoma [35, 36].

In a similar fashion, we detected variation on different genes associated with CLL evolution. The most relevant was the association of *RIP3K* with both time to treatment and overall survival. *RIP3K* encodes a protein that regulates necroptosis, a form of regulated cell death characterized by cell membrane permeabilization [37]. Other relevant genes associated with rapid progression were the pro-proliferative gene *NIFK* [38], the tumor suppressor *SIK1* [39, 40] and *ZCCHC7*, which is encoded near the B-cell specific PAX5 super-enhancer locus [41, 42]. On the other hand, various genes were associated with overall survival, such as *CLUAP1*, which participates in tumor growth and cytoskeleton regulation [43–45]; and the enzyme *GAMT*, which converts S-adenosylmethionine to creatine in order to foster high energy demands [46]. Moreover, the BCL-2 interacting gene *BNIPL* [47, 48] was suggestively associated with survival and deserves further characterization. In the same direction, the most remarkable pathway-level association with survival was that of the pentose phosphate metabolic pathway, which fuels cells with metabolites for nucleotide and lipid biosynthesis, and provides reducing power to promote cell survival under stressful conditions [49]. Other pathways such as GNα13 and Nitric Oxide were also significant. Concordantly, recurrent inactivating mutations in the G-protein superfamily gene *GNA13* have been described in B cell lymphomas [50–53], and the contribution of nitric oxide to apoptosis resistance in CLL cells has been addressed by various studies [54, 55].

The main limitation of this study is the lack of an independent cohort for validation of these findings. Furthermore, although inflation values were low, we assume that treatment heterogeneity could have an impact on overall survival associations. Nevertheless, the global results are not only statistical significant but also biologically plausible. Thus, we believe that this report will motivate further studies in order to confirm the effect of these variants and to determine their mechanisms of action in lymphoproliferative disorders.

## Conclusions

Our results point towards the existence of germline variability as a determinant of CLL clinical aggressivity. Future studies to validate and characterize the activity of these variants in CLL are needed.

## Additional files

Additional file 1: **Figure S1.** Principal component plots for the subjects included in the final analysis. (JPG 154 kb)

Additional file 2: **Figure S2.** Manhattan plot of the additive OS model results. (JPG 199 kb)

Additional file 3: **Figure S3.** Manhattan plot of the dominant OS model results. (JPG 222 kb)

Additional file 4: **Figure S4.** Manhattan plot of the recessive TTT model results. (JPG 193 kb)

Additional file 5: **Figure S5.** Manhattan plot for the Pentose Phosphate pathway. (JPG 56 kb)

Additional file 6: **Figure S6.** Manhattan plot for the GNα13 pathway. (JPG 54 kb)

Additional file 7: **Figure S7.** Manhattan plot for the *“Induction of apoptosis by extracellular signal”* biological process. (JPG 59 kb)

Additional file 8: **Figure S8.** Manhattan plot for the *“Mitosis”* biological process. (JPG 54 kb)

Additional file 9: **Table S1.** GWAS results of the additive Cox regression model for TTT. Polymorphisms with a BH-adjusted *P*-value < 0.5 are shown. **Table S2.** GWAS results of the dominant Cox regression model for TTT. Polymorphisms with a BH-adjusted *P*-value < 0.5 are shown. **Table S3.** GWAS results of the recessive Cox regression model for TTT. Polymorphisms with a BH-adjusted *P*-value < 0.5 are shown. **Table S4.** GWAS results of the additive Cox regression model for OS. Polymorphisms with a BH-adjusted *P*-value < 0.5 are shown. **Table S5.** GWAS results of the dominant Cox regression model for OS. Polymorphisms with a BH-adjusted *P*-value < 0.5 are shown. **Table S6.** GWAS results of the recessive Cox regression model for OS. Polymorphisms with a BH-adjusted *P*-value < 0.5 are shown. **Table S7.** *VEGAS2* gene-level results for association with TTT. **Table S8.** *VEGAS2* gene-level results for association with OS. (XLSX 3081 kb)

## Abbreviations

BH: Benjamini-Hochberg
CLL: Chronic Lymphocytic Leukemia:
EGA: European Genome-Phenome Database
IBD: Identity By Descent
ICGC: International Cancer Genome Consortium
LD: Linkage Disequilibrium
MBL: Monoclonal B cell lymphocytosis
OS: Overall Survival:
PCA: Principal Component Analysis
TTT: Time to Treatment
VAF: Variant allele frequency
