# Supplementary figures and images for: The association of germline variants with chronic lymphocytic leukemia outcome suggests the implication of novel genes and pathways in clinical evolution
Source: BMC Cancer. 2019 May 29;19:515. doi: 10.1186/s12885-019-5628-y (PMC6542042; doi:10.1186/s12885-019-5628-y)

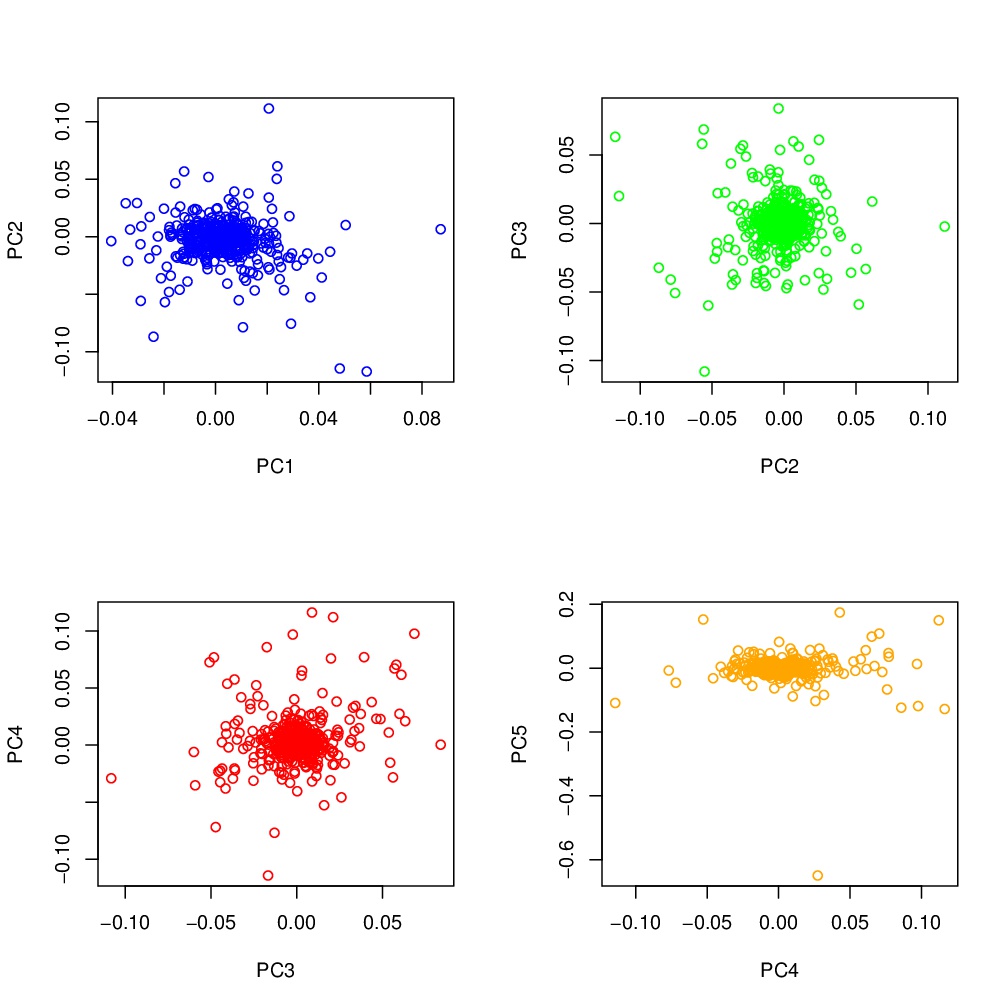

Supplement: Supplementary file 1 — Figure S1. Principal component plots for the subjects included in the final analysis. (JPG 154 kb) [file 12885_2019_5628_MOESM1_ESM.jpg]

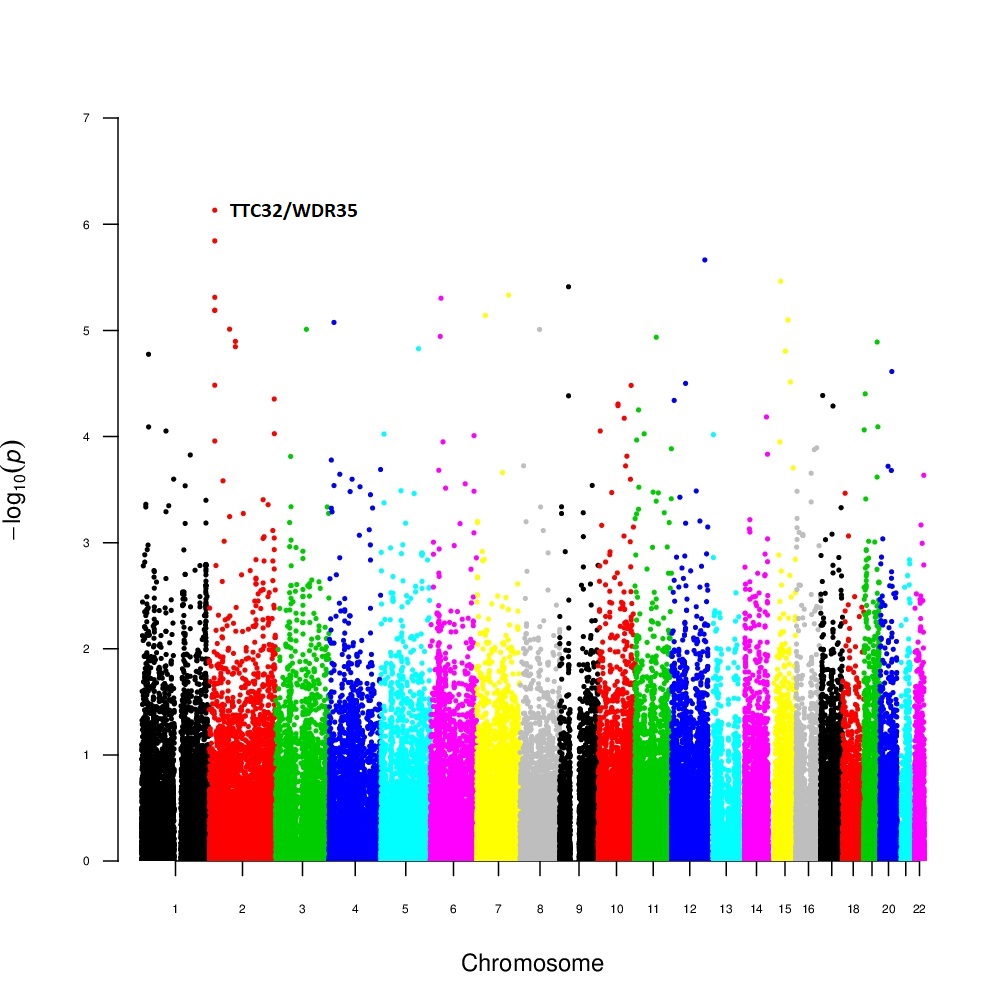

Supplement: Supplementary file 2 — Figure S2. Manhattan plot of the additive OS model results. (JPG 199 kb) [file 12885_2019_5628_MOESM2_ESM.jpg]

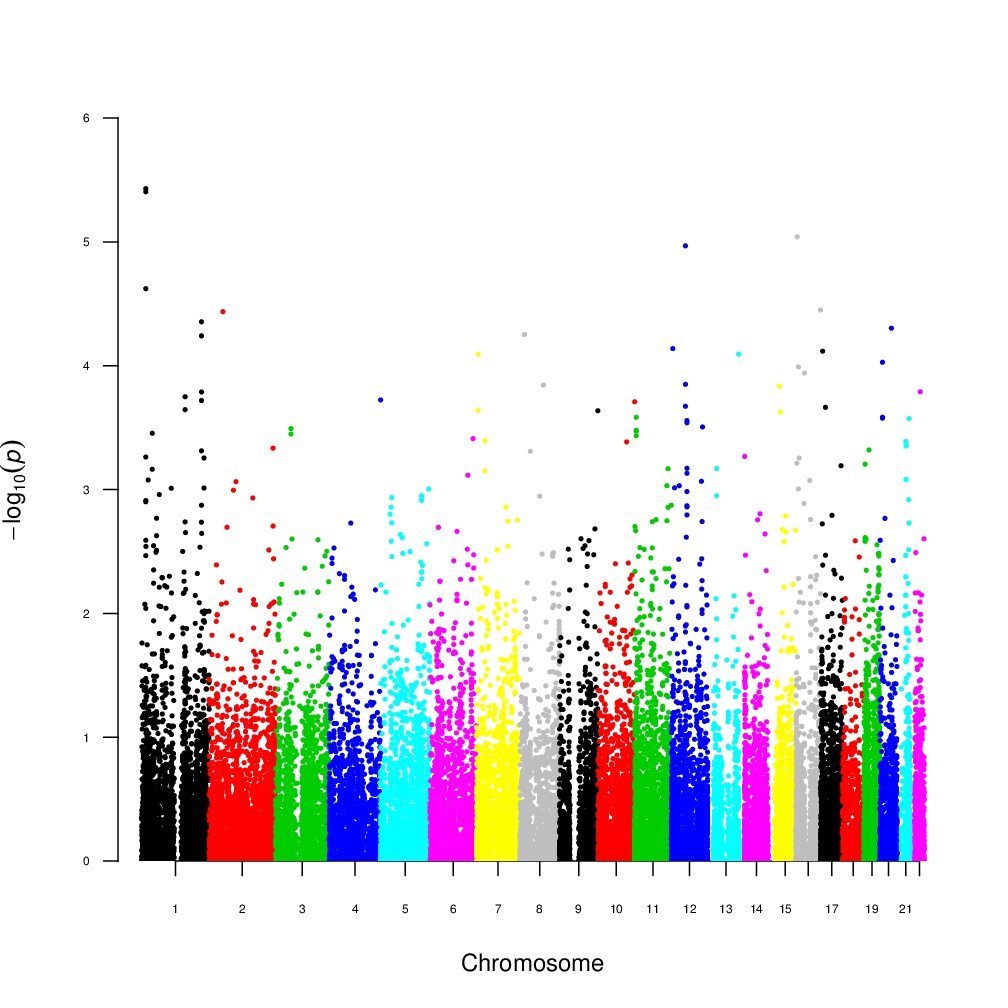

Supplement: Supplementary file 3 — Figure S3. Manhattan plot of the dominant OS model results. (JPG 222 kb) [file 12885_2019_5628_MOESM3_ESM.jpg]

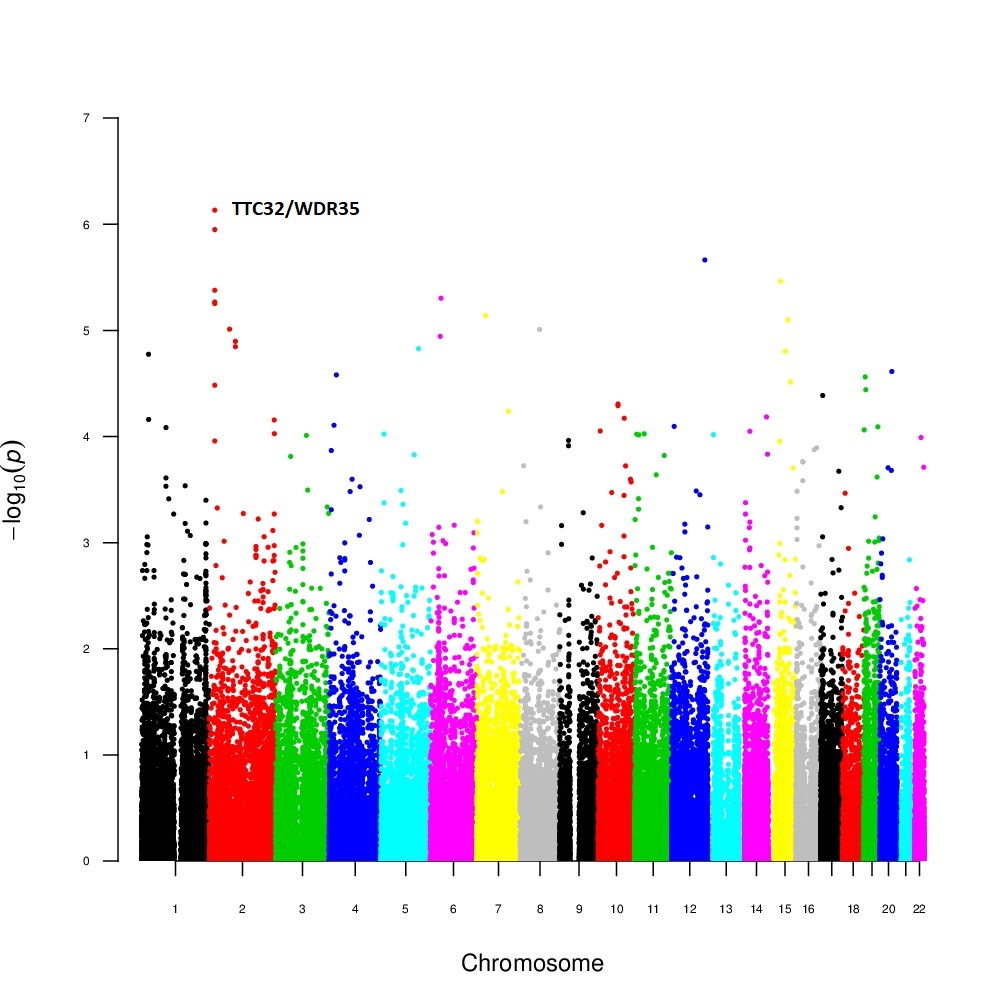

Supplement: Supplementary file 4 — Figure S4. Manhattan plot of the recessive TTT model results. (JPG 193 kb) [file 12885_2019_5628_MOESM4_ESM.jpg]

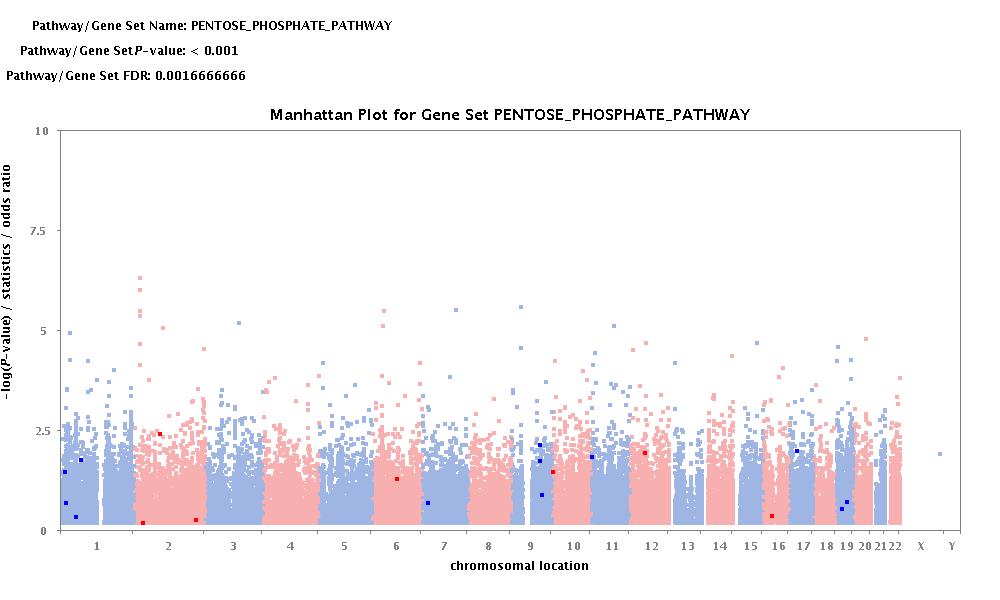

Supplement: Supplementary file 5 — Figure S5. Manhattan plot for the Pentose Phosphate pathway. (JPG 56 kb) [file 12885_2019_5628_MOESM5_ESM.jpg]

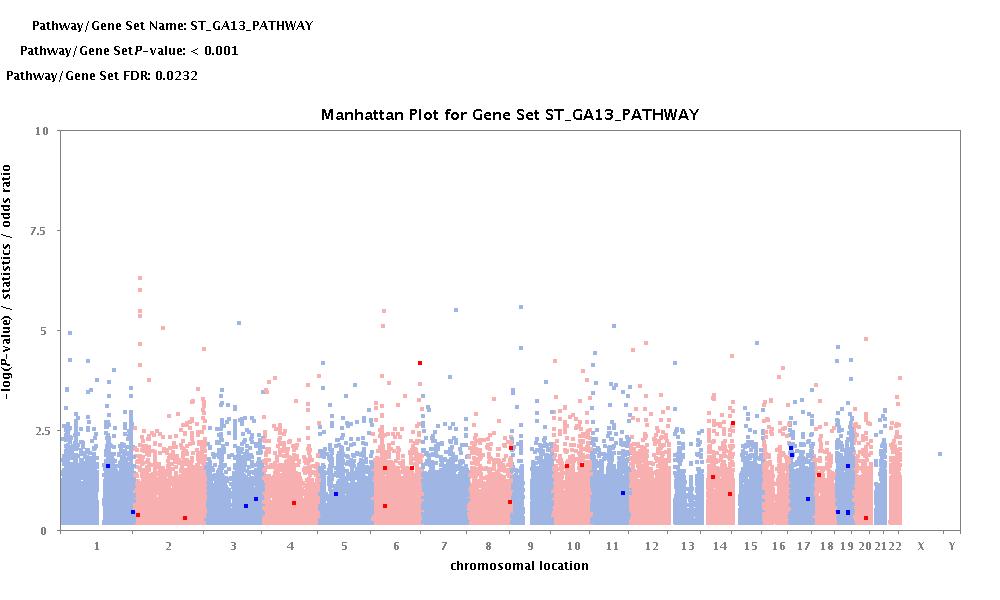

Supplement: Supplementary file 6 — Figure S6. Manhattan plot for the GNα13 pathway. (JPG 54 kb) [file 12885_2019_5628_MOESM6_ESM.jpg]

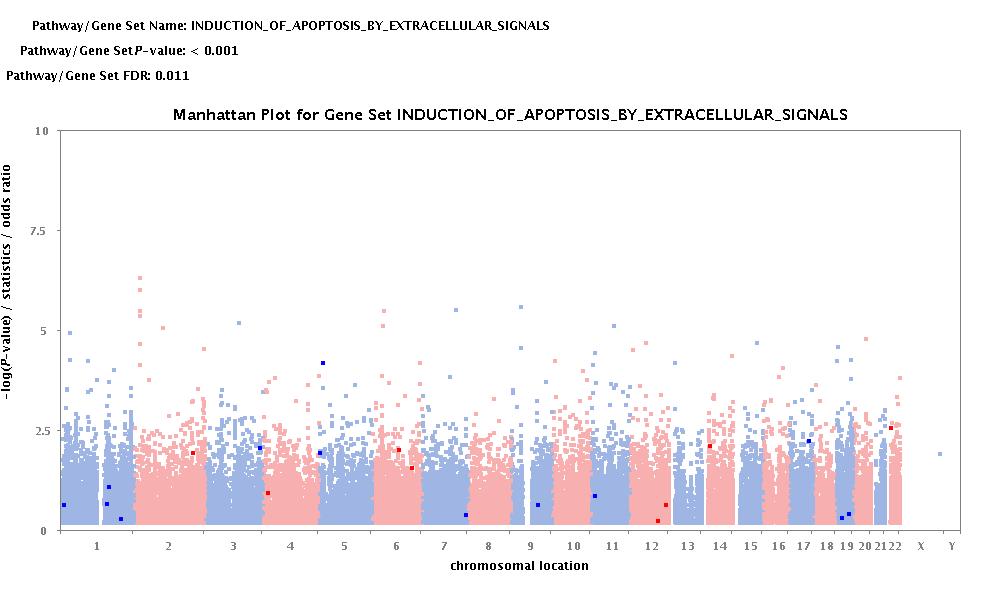

Supplement: Supplementary file 7 — Figure S7. Manhattan plot for the “Induction of apoptosis by extracellular signal” biological process. (JPG 59 kb) [file 12885_2019_5628_MOESM7_ESM.jpg]

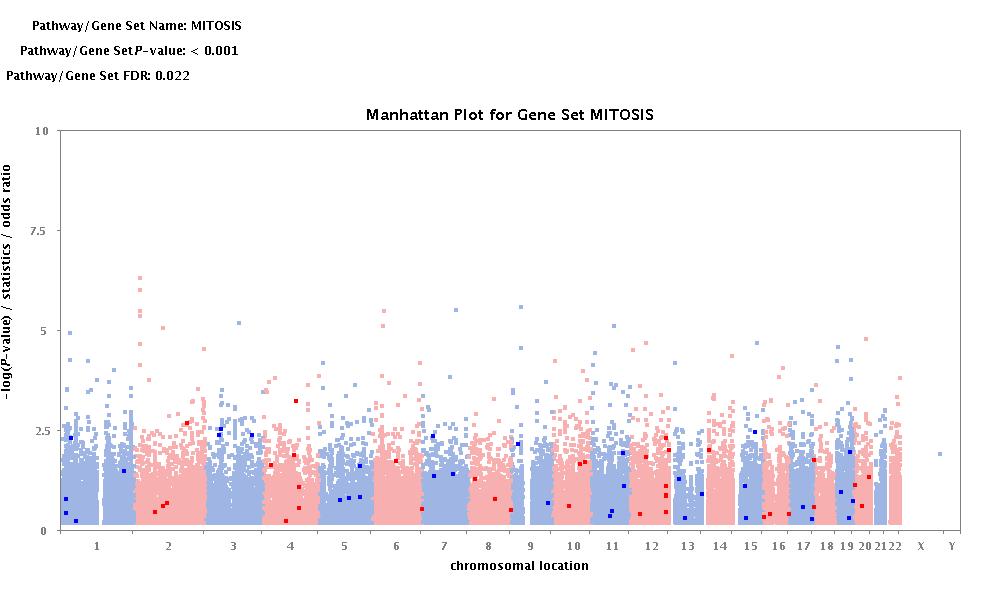

Supplement: Supplementary file 8 — Figure S8. Manhattan plot for the “Mitosis” biological process. (JPG 54 kb) [file 12885_2019_5628_MOESM8_ESM.jpg]
